# Do recommended interventions widen or narrow inequalities in musculoskeletal health? An equity-focussed systematic review of differential effectiveness

**DOI:** 10.1093/pubmed/fdac014

**Published:** 2022-03-06

**Authors:** G Peat, K P Jordan, R Wilkie, N Corp, D A van der Windt, D Yu, G Narle, N Ali

**Affiliations:** Primary Care Centre Versus Arthritis, School of Medicine, Keele University, Keele, Staffordshire, ST5 5BG, UK; Primary Care Centre Versus Arthritis, School of Medicine, Keele University, Keele, Staffordshire, ST5 5BG, UK; Primary Care Centre Versus Arthritis, School of Medicine, Keele University, Keele, Staffordshire, ST5 5BG, UK; Primary Care Centre Versus Arthritis, School of Medicine, Keele University, Keele, Staffordshire, ST5 5BG, UK; Primary Care Centre Versus Arthritis, School of Medicine, Keele University, Keele, Staffordshire, ST5 5BG, UK; Primary Care Centre Versus Arthritis, School of Medicine, Keele University, Keele, Staffordshire, ST5 5BG, UK; Public Health England, London, SE1 8UG, UK; Versus Arthritis, Chesterfield, S41 7TD, UK; Office for Health Improvement and Disparities, Department of Health and Social Care, London, SW1H 0EU, UK

**Keywords:** musculoskeletal disorders, social stratifiers, health inequalities, health outcomes, interventions, systematic review

## Abstract

**Background:**

It is unclear whether seven interventions recommended by Public Health England for preventing and managing common musculoskeletal conditions reduce or widen health inequalities in adults with musculoskeletal conditions.

**Methods:**

We used citation searches of Web of Science (date of ‘parent publication’ for each intervention to April 2021) to identify original research articles reporting subgroup or moderator analyses of intervention effects by social stratifiers defined using the PROGRESS-Plus frameworks. Randomized controlled trials, controlled before-after studies, interrupted time series, systematic reviews presenting subgroup/stratified analyses or meta-regressions, individual participant data meta-analyses and modelling studies were eligible. Two reviewers independently assessed the credibility of effect moderation claims using Instrument to assess the Credibility of Effect Moderation Analyses. A narrative approach to synthesis was used (PROSPERO registration number: CRD42019140018).

**Results:**

Of 1480 potentially relevant studies, seven eligible analyses of single trials and five meta-analyses were included. Among these, we found eight claims of potential differential effectiveness according to social characteristics, but none that were judged to have high credibility.

**Conclusions:**

In the absence of highly credible evidence of differential effectiveness in different social groups, and given ongoing national implementation, equity concerns may be best served by investing in monitoring and action aimed at ensuring fair access to these interventions.

## Background

A substantial proportion of disability, sickness absence and lost productivity are attributed to musculoskeletal disorders.^1^^,^^2^ In the UK, they account for 12–14% of primary care consultations in adults^3^^,^^4^ and a significant proportion of healthcare expenditure.^5^ Low back pain, neck pain, osteoarthritis and other non-inflammatory painful disorders that are common across the adult life course dominate this picture. These conditions tend to be more frequent and disabling in socially disadvantaged groups.^6–10^ The need to systematically scale up and implement ‘high-value’ interventions and models of care is a priority^11–13^ and is a specific component of the national strategic framework recently published by Public Health England, NHS England and Versus Arthritis.^14^ However, within this framework there must be due regard to health inequalities. Interventions that improve the health of a population overall may have no effect on reducing inequalities: some may even increase inequalities (so-called ‘intervention-generated inequalities’).^15^ In theory, inequalities can be introduced, abolished or modified at multiple stages in the provision of, and response to, intervention.^15^^,^^16^ In this review we focus on inequalities in the effectiveness of interventions to prevent and manage common musculoskeletal disorders, i.e. differences in the response to interventions between socially advantaged and disadvantaged groups who have gained access to these interventions.

### Interventions recommended in Public Health England’s Return on Investment tool

The return on investment (RoI) tool for local authorities and healthcare commissioners^17^ used a combination of stakeholder groups, literature review and economic modelling to prioritize seven clinical- and cost-effective interventions for high-volume musculoskeletal conditions in working age adults. These are: cognitive–behavioural therapy (CBT)/psychological approaches including exercise,^18^^,^^19^ stratified risk assessment and care^20^ and Yoga for Healthy Lower Backs^21^ for low back pain; Enabling Self-Management and Coping of Arthritic Knee Pain Through Exercise (ESCAPE-pain) for knee pain/osteoarthritis^22^; Physiotherapist-led telephone assessment and treatment service (PhysioDirect) early telephone assessment and advice,^23^ self-referral to NHS physiotherapy^24^^,^^25^ and vocational advice in primary care^26^ for all musculoskeletal disorders. The content of each intervention as originally trialled is summarized in Supplementary Data Table S1.^18–37^ These disparate interventions are each complex and span individual- and group-level, therapist-delivered interventions and alternative models of health service organization. There is no simple explanation of how each ‘works’ and, by extension, how differential outcomes might plausibly arise among socially defined groups accessing the intervention. CBT, for example, can encompass a wide range of cognitive and behavioural techniques,^38^^,^^39^ which plausibly operate through both general and specific mechanisms.^40^^,^^41^ Mediation analyses of the Back Skills Training (BeST) intervention suggest that improving self-efficacy and reducing fear avoidance may be important mechanisms of action.^40^ Similar analyses of the stratified care approach known as STarT Back have found that reducing pain and psychological distress are important mechanisms.^41^ It is unclear whether such mechanisms would inherently favour more socially advantaged groups, but it has been proposed that intervention-generated inequalities are more likely for ‘downstream’ interventions that target individual behaviour change and require high levels of personal agency.^42–44^ None of the interventions recommended in the RoI tool were deliberately designed to reduce health inequalities. In this context it is possible that socially disadvantaged groups may gain less from these interventions. The aim of our review was to identify and critically synthesize available evidence on whether socially disadvantaged groups, defined using the PROGRESS-Plus framework,^45^^,^^46^ benefit more or less than their more advantaged counterparts from these interventions once they have gained access to them.

## Methods

### Protocol registration

The protocol for this review was registered on PROSPERO (CRD42019140018).

### Search strategy

As our review was concerned with evidence of differential response for a defined set of published interventions, our search strategy focussed on searching for citations of key references (‘parent publications’^47^). Parent publications for each of the seven interventions included journal articles and funder reports of the main clinical and cost-effectiveness findings as well as published protocols for the original trial. Using a total of 20 parent publications^18–37^ we conducted a ‘Cited Reference Search’ in Web of Science for ‘child’ publications reporting relevant evidence of differential response to treatment by social stratifiers. An initial scoping search was performed to judge the possible yield from this strategy. The citation search covered the period from the date of publication of each ‘parent publication’ to 13 April 2021 with no language restrictions.

### Eligibility criteria and study selection

Social stratifiers were hypothesized to be predictors of differential effect of intervention (also referred to as ‘effect modifiers’ or ‘treatment moderators’).^48^^,^^49^ The term ‘differential’ signals the need for comparative evidence on the benefits and harms from one intervention compared with another. The comparators could be a different intervention, a different dose of the same intervention or no intervention. We included randomized controlled trials (RCT), including cluster RCTs and stepped-wedge designs, controlled before-after studies and interrupted time series. Systematic reviews presenting subgroup, stratified analyses or meta-regressions, as well as individual participant data (IPD) meta-analyses and modelling studies were also eligible. Studies had to present data on differences in intervention effectiveness between groups defined by a PROGRESS-Plus social stratifier (place of residence; race/culture/ethnicity/language; occupation; gender/sex; religion; employment; socioeconomic position; social capital; other protected characteristics and vulnerable groups, e.g. age). Patient outcomes of interest included health status (e.g. pain, disability), health behaviours, healthcare costs, quality-adjusted life years (QALY), cost per QALY and work outcomes (e.g. sickness absence, lost productivity).

We excluded studies and reviews that included only observational data on inequalities in health status or access to interventions, reported outcomes of interventions targeted at a particular social group without a suitable PROGRESS-Plus comparator, had total sample size <100. Studies had to be of adults aged 18 years and over with a relevant musculoskeletal condition (e.g. low back pain for BeST, STarT Back and Yoga for Healthy Lower Backs) and not restricted only to patients with inflammatory disease or trauma/injury. Protocol papers, editorials, correspondence, conference abstracts and non-English language articles were excluded.

Screening of titles and abstracts and review of full-text articles for inclusion were conducted by two independent reviewers with disagreement resolved by consensus, using Rayyan^50^ to manage this process.

### Data extraction

Data extraction was conducted by one reviewer using a form that we designed before data extraction began. Data extraction was checked by a second reviewer resolving discrepancies through discussion. In addition to standard descriptive fields, we extracted information on (i) baseline distribution of PROGRESS-Plus characteristics; (ii) methods, results (e.g. absolute or standardized mean differences between social stratifier groups) and author conclusions on moderator analyses by each PROGRESS-Plus variable; (iii) selective participation or attrition by PROGRESS-Plus characteristics.

### Quality assessment/risk of bias

We assessed the credibility of potentially relevant treatment effect moderation reported in analyses of a single RCT or in meta-analysis of multiple RCTs using the Instrument to assess the Credibility of Effect Moderation Analyses (ICEMAN).^51^ The CHecklist for the Appraisal of Moderators and Predictors^52^ would have been equally suitable. Both provide structured appraisal tools derived through a rigorous development process including expert consensus. We did not assess risk of bias in the main effects of the trials but instead referred to risk of bias assessments reported in previous systematic reviews.

### Data synthesis

Given heterogeneity in populations, interventions and outcomes, we anticipated a narrative synthesis approach rather than formal meta-analysis. Where available, we sought to summarize both relative and absolute differences in effectiveness of interventions by social stratifier.

## Results

### Study selection

**Fig. 1:**
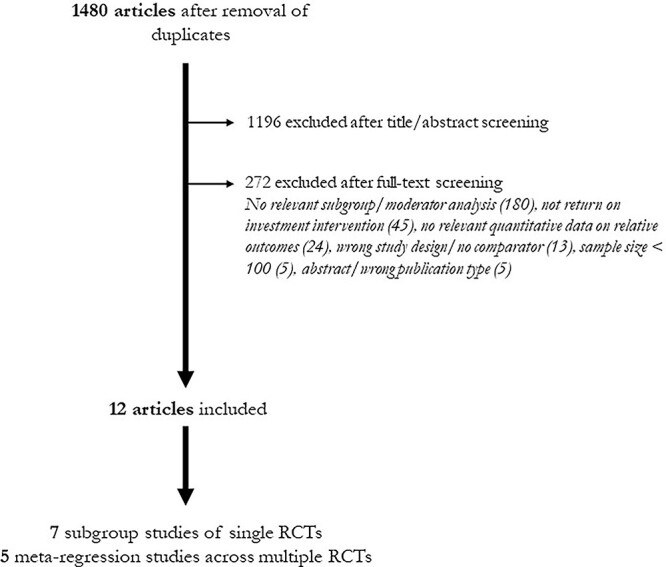
Flowchart of studies.

The citation search yielded 1480 potentially relevant articles after removal of duplicates, of which 12 were included (Fig. 1; Supplementary Data Table S2).

### Study characteristics

Six of the 12 included studies were moderator (subgroup) analyses of data from single RCTs, typically the original trial, of the BeST,^27^^,^^53^^,^^54^ STarT Back^55^ and PhysioDirect^23^^,^^36^ interventions. We included a secondary analysis of BeST trial data^56^ estimating the additional effect of the intervention among treatment compliers which related this to social stratifiers (Table 1). The remaining five studies were conventional meta-analyses that included at least one trial of an intervention in the PHE RoI tool^57–61^ (Table 2). No relevant moderator analyses by social stratifiers were found for self-referral to physiotherapy or vocational advice in primary care.

### Evidence of differential effect, by intervention

#### CBT, including exercise

Within the economic analysis of the BeST trial data,^27^ women had slightly lower incremental costs and slightly higher incremental QALYs than men, resulting in 30-40% lower incremental cost-effectiveness ratio (ICER) estimates. Compared with younger ages, older adults (>60 years) were estimated to have higher incremental QALYs but also much higher incremental costs, resulting in 20–70% higher ICER estimates. ICER values for all subgroups were well below established thresholds for cost-effectiveness.

In addition to pre-specified moderator analyses of the BeST trial, Underwood *et al*.^53^ undertook additional exploratory moderator analyses including several variables related to PROGRESS-Plus domains (Table 1). After adjustment for potential confounders, only interaction terms with age and employment status reached statistical significance, with improvement in disability scores, but not pain and other functional outcomes, favouring younger and employed participants.

Using latent class analysis of baseline psychological and symptom severity variables in the BeST trial, Barons *et al*.^54^ extracted three subgroups of patients. These subgroups differed with respect to their age distribution, proportion of women and employment status. In a model predicting recovery (defined as change in disability score at 12 months), the interaction terms between treatment and subgroup were statistically non-significant. However, an interaction of treatment with work indicated there may be better outcomes of the intervention in those in work.

In a complier average causal effects analysis of the BeST trial data, Knox *et al*.^56^ estimated that the effect on disability score at 12 months of intervention was slightly greater among treatment compliers. Non-compliers tended to be younger, but there was no statistically significant difference in treatment compliance by gender, ethnicity, education or employment status.

Bernard *et al*.^59^ conducted a series of bivariate random-effects meta-regression models using data from studies of CBT with exercise in chronic diseases (Table 2). The only statistically significant moderation effect by age or gender was a larger effect on fatigue for women compared with men. This specific analysis did not include the ‘parent’ trial by Johnson *et al*.

Martinez-Calderon *et al.*^61^ found no statistically significant moderation of effect by age on self-efficacy outcomes up to 12 months after psychological therapies, including BeST.

**Table 1:** Moderator analyses of individual trials

**Authors, year, country**	**RoI Intervention and comparator**	**Population**	**Study size**	**Outcomes**	**Moderators investigated**	**Findings**	**Authors interpretations/conclusions**
Lamb, 2010, England^27^	Group CBT (BeST) versus Active management advisory consultation	Adults (aged ≥18 years) with at least moderately troublesome subacute or chronic low back pain	528	Incremental costs and QALYs from NHS and general healthcare perspectives	Patient age, sex	ICER (NHS perspective): Male = £2422, Female = £1461; Age > 60 = £1855, Age ≤ 60 = £1538. ICER (general healthcare perspective): Male = £3912, Female = £2657; Age > 60 = £3692, Age ≤ 60 = £2185.	The subgroup analysis reported here shows confirmatory evidence that age, sex and duration of back pain do not have a large impact on the cost-effectiveness of CBT, which remains well below currently recognized cost-effectiveness thresholds.
Underwood, 2011, England^53^	Group CBT (BeST) versus Active management advisory consultation	Adults (aged ≥18 years) with at least moderately troublesome subacute or chronic low back pain	598	Pain and disability (ΔRMDQ, ΔMVK Pain, ΔMVK Disability) at 12 months	Patient age, sex, educational level, employment status, receipt of benefits, ethnicity	Covariate-adjusted mean difference in estimated treatment effect between subgroups all statistical non-significant at *P* = 0.05 except for RMDQ (positive value indicates greater benefit): Age ≥ 54 versus < 54 = −1.58 (−3.05, −0.12); Employed versus not employed = 1.89 (0.43, 3.35).	The only moderation by baseline variables of the effect of randomization was on the RMDQ outcome. Being younger and currently working both moderated treatment effect, resulting in larger response to treatment.
Barons, 2014, England^54^	Group CBT (BeST) versus Active management advisory consultation	Adults (aged ≥18 years) with at least moderately troublesome subacute or chronic low back pain	407	Low back pain-related functional limitation (ΔRMDQ≥3) at 12 months	Participants grouped based on baseline psychological and symptom severity variables (latent classes) which differed with respect to age, sex, and employment status; also examined work separately	No significant interaction between class and outcome. Interaction of treatment with work *P* = 0.02—treatment more effective in those in work	For the trial participants who received the intervention, there was an association between class membership and outcome, but not for those who did not receive the intervention. However, we were unable to detect an effect on outcome from interaction between class membership and receiving the intervention or not. Predictive effect of improving with treatment for those in work.
Knox, 2014, England^56^	Group CBT (BeST) versus Active management advisory consultation	Adults (aged ≥18 years) with at least moderately troublesome subacute or chronic low back pain	598	Treatment compliance (and effect of compliance on pain, disability, and health-related quality of life (ΔRMDQ, ΔMVK Pain, ΔMVK Disability, EQ-5D) at 12 months)	Patient age, sex, educational level, employment status, ethnicity	Mean compliance difference (positive indicates better outcome in compliers): RMDQ = 0.4 (−0.6, 1.5); MVK Pain = 6.0 (0.8, 11.1); MVK Disability = 2.8 (−2.1, 7.7); EQ-5D = 0.01 (−0.04, 0.07). Associations with compliance: age (*P* = 0.001), sex (*P* = 0.69), educational level (*P* = 0.58), employment status (*P* = 0.61), ethnicity (*P* = 0.26)	Treatment compliance is important in the effectiveness of group cognitive behavioural intervention. Younger people... are at greater risk of non-compliance.
Beneciuk, 2017, England^55^	Stratified primary care management (STarT Back) versus Non-stratified current best practice	Adults (aged ≥18 years) consulting for back pain (with or without radiculopathy)	688	Low back pain-related functional limitation (RMDQ≥7) at 4 months	Patient age group, sex, educational level, SES (occupational class), employment status	Interaction terms: Age, *P* = 0.68; Sex, *P* = 0.89; Educational level, *P* = 0.109; Occupational class, *P* = 0.03 (stratified care: better outcome with high SES); Employment status, *P* = 0.68	SES was identified as an effect modifier for disability outcomes with education level meeting criteria for effect modification with weaker evidence. We have provided preliminary exploratory findings about characteristics of patients who might least likely benefit from prognostic stratified care treatment for low back pain.
Salisbury, 2013a^23^, 2013b^36^, England	Direct access to telephone consultation with a physiotherapist, followed by face-to-face physiotherapy if necessary versus Usual care	Adults (aged ≥18 years) referred by GPs or self-referred for musculoskeletal physiotherapy	1912	Physical health (SF-36v2 PCS) at six months	Patient age group, patient SES (area-level deprivation)	Interaction terms: Age, *P* = 0.59; Deprivation, *P* = 0.62	No evidence was found of subgroup differences for the primary outcome. However, potentially important differences cannot be excluded…

EQ-5D EuroQol; MVK Modified Von Korff; RMDQ Roland-Morris Disability Questionnaire; Δ change (in score from baseline to follow-up).

**Table 2:** Moderator analyses across multiple trials

**Authors, year**	**Interventions**	**Populations**	**Study size**	**Outcomes**	**Moderators investigated**	**Findings**	**Authors interpretations/conclusions**
Kroon, 2014^57^	Structured self-management education programmes versus attention control, usual care, information alone or other intervention^a^	People diagnosed with OA	12 studies (total n = 3188)	1. Self-management of OA, 2. Participant’s positive and active engagement in life, 3. Pain, 4. Global OA scores, 5. Self-reported function, 6. Quality of life, 7. Withdrawals.	Study populations classed as mainly Caucasian, educated females versus not	SMD (Self-management of OA) = −0.29 (0.07, 0.50) versus 0.03 (−0.29, 0.36), *P* = 0.20. SMD (Function) = −0.20 (−0.37, −0.02) versus −0.06 (−0.21, −0.01), *P* = 0.26. SMD (Pain) = −0.11 (−0.30, 0.07) versus −0.20 (−0.35, −0.05), *P* = 0.49. RR (Withdrawals) = 1.05 (0.76, 1.45) versus 1.04 (0.63, 1.69), *P* = 0.97	Self-management programmes appeared more beneficial for Caucasian, educated female participants with respect to self-management of OA and self-reported function, but for self-reported pain, self-management programmes appeared more beneficial in trials that did not primarily include this subgroup.
Niknejad, 2018^58^	Psychological interventions using CBT alone or in combination versus control (various)^a^	Adults with chronic pain, focused on older individuals (sample mean age of ≥60 years) (17 of 22 studies musculoskeletal)	22 studies (total *n* = 2608)	1. Pain, 2. Psychological, 3. Function	Mean age of sample, proportion of women in sample	Statistically non-significant (estimates and *P*-values not reported).	Across all outcomes and possible moderators, only mode of therapy showed a coherent pattern of results. Other moderators were nonsignificant, and there were no indications of negative results for any subgroup.
Bernard, 2018^59^	CBT combined with physical exercise versus usual care, wait-list, or an active comparison control^b^	Adults with chronic disease (7 of 30 studies musculoskeletal)	30 studies (*n* = 30-555 per study)	1. Depression, 2. Anxiety, 3. Fatigue, 4. Pain	Mean age of sample, proportion of women in sample	Only interaction reported as significant was for gender with fatigue (β = −0.62; 95% CI [−1.17, −0.08]; *P* = 0.03). Others interactions assumed not statistically significant.	For fatigue, women participants had more benefits fromCBTEx interventions.
Zou, 2019^60^	Mindful exercise (e.g. Tai Chi, Qigong, Yoga)^c^	Adults with chronic low back pain	17 studies (total *n* = 2022)	1. Pain intensity, 2. Disability	Mean age of sample	Statistically non-significant (estimates and *P*-values reported in main text)	No significant differences were observed.
Martinez-Calderon, 2020^61^	Exercise^c^; self-management; psychological therapy^d^; multicomponent intervention	Adults with chronic musculoskeletal pain	60 studies (total *n* = 12415)	Self-efficacy at 0–3, 3–6 months	Age	Statistically non-significant (estimates and *P*-values reported in appendix)	Age did not moderate the effects of any intervention.

OA, osteoarthritis; RR, risk ratio; SMD, standardized mean difference.

^a^Includes ESCAPE-pain^22^

^b^Includes Johnson *et al.*^18^

^c^Includes Tilbrook *et al*.^21^

^d^Includes Lamb *et al*.^19^

#### Stratified care for low back pain (STarT back)

In a secondary analysis of STarT Back trial data, Beneciuk *et al.*^55^ used univariable selection followed by a series of logistic regression models adjusting for baseline disability score and including an interaction term to explore nine potential moderators of treatment effect, including age, sex, educational level, socioeconomic status (SES, defined by individual occupational class) and employment status, on poor disability outcome at 4 months. The interaction term for occupational class was statistically significant (*P* = 0.028) with the beneficial effect of the STarT Back intervention greatest among higher SES participants and least in lower SES participants. This is suspected to be due to a lack of effect of promoting self-management without further face-to-face physiotherapist support in lower SES patients with low health literacy (personal correspondence, J. Hill). The interaction term for educational level was in the same direction, suggesting greater benefit among more educated participants, but was statistically non-significant (*P* = 0.109). Interaction terms for age, sex and employment status were non-significant (*P* > 0.20).

#### Yoga for healthy lower backs

Meta-regression analyses by Zou *et al.*^60^ and Martinez-Calderon *et al.*,^61^ found no statistically significant moderation of effect by age. Zou *et al*.’s review included Yoga for Healthy Lower Backs trial findings alongside other forms of mindful exercise for chronic low back pain on the outcomes of pain intensity and disability. Martinez-Calderon *et al.*’s included various exercise intervention trials for low back pain with a focus on self-efficacy outcomes up to 12 months after intervention.

#### Physiotherapist-led telephone assessment and treatment service

In pre-specified moderator analyses Salisbury *et al.*^23^^,^^35^ found no moderation of treatment effect by patient age or neighbourhood deprivation. In the context of a trial powered for, and finding, equivalence in clinical effectiveness, the detection of strong subgroups could not be expected. No moderator analyses were reported for secondary outcomes or cost-effectiveness.

#### ESCAPE-pain

Meta-regression analyses by Niknejad *et al.*^58^ found that the effects on pain, psychological outcomes or function of psychological interventions using CBT alone or in combination (e.g. with exercise) in chronic pain did not appear to vary according to the mean age or proportion of women in each study. It was unclear whether the original trial of ESCAPE-pain was included in those analyses.

In contrast, Kroon *et al.*^57^ concluded that the effects of self-management programmes for people with osteoarthritis appeared greater in studies with predominantly educated, Caucasian women, although the findings were sensitive to the particular outcome of interest. The original ESCAPE-pain trial was not included in those analyses.

### Credibility of claims of treatment effect moderation

We applied ICEMAN^51^ to the potentially relevant findings of treatment effect moderation reported by Underwood *et al.*,^53^ Barons *et al.*,^54^ Beneciuk *et al.*,^55^ and Kroon *et al*.^57^ Previous systematic reviews^62^^,^^63^ judged the BeST, STarT Back and ESCAPE-pain trials to be at low risk of bias for main effects, although attrition bias was highlighted as a potential risk in the latter. We judged all such findings to have either low or very low credibility, with the exception of the finding that participants from lower SES may benefit less from STarT Back (in terms of self-reported disability at 4 months) than those from higher SES. This finding was judged to have moderate credibility (Table 3).

**Table 3:** Evaluation of credibility of findings of differential effect

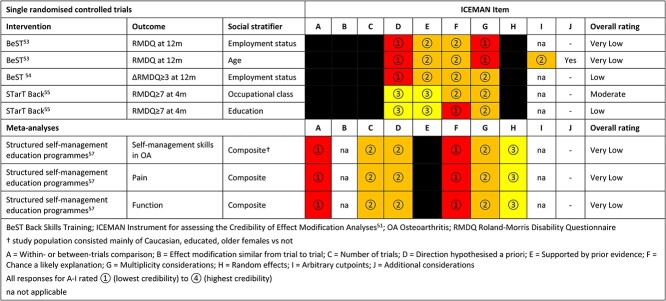

ICEMAN Instrument for assessing the Credibility of Effect Modification Analyses^51^; RMDQ Roland–Morris Disability Questionnaire.

A = Within- or between-trials comparison; B = Effect modification similar from trial to trial; C = Number of trials; D = Direction hypothesized a priori; E = Supported by prior evidence; F = Chance a likely explanation; G = Multiplicity considerations; H = Random effects; I = Arbitrary cutpoints; J = Additional considerations

All responses for A-I rated ① (lowest credibility) to ④ (highest credibility)

na, not applicable

^a^Study population consisted mainly of white, educated, older females versus not

## Discussion

### Main finding of this study

Theoretically informed, pre-specified subgroup analyses of RCT data, adequately powered and appropriately conducted and reported, provide the best available evidence of differential effectiveness of interventions. We found no claims of differential effectiveness of interventions recommended in the PHE ROI tool that met this high bar of evidence. Some evidence that low back pain (LBP) patients of lower occupational class might benefit less from the STarT Back stratified care intervention was judged as moderately credible. However, this was not a pre-specified subgroup analysis in the original trial and, unlike secondary analyses of the BeST intervention,^53^ corroboration of this finding across more than one outcome was not attempted. We found no studies that had clearly assessed moderating effects of social stratifiers for PhysioDirect, self-referral to physiotherapy, ESCAPE-pain or vocational advice in primary care.

### What is already known on this topic

The absence of highly credible, ‘confirmatory’ evidence of differential effectiveness of interventions is not unexpected: a similar conclusion was reached in previous reviews in the general medical literature^64^^,^^65^ and of low back pain interventions.^66^ While reviews seldom focus exclusively on social disadvantage, many of the challenges, such as inadequate statistical power, are relevant irrespective of which subgroups are of interest, and were noted by Inglis *et al*.^67^ in their review of public health interventions. Conventional meta-regression analysis arguably adds little, given its low power to detect differences and susceptibility to study-level confounding.^68^^,^^69^ In published systematic reviews, analyses of differential effectiveness of interventions by age, sex or race/ethnicity are rarely planned, seldom undertaken and may rest upon a single trial without subsequent corroboration.^70–73^ IPD meta-analysis offers advantages but can take considerable resource to pool data and comparable information on SES may not be available.^74^^,^^75^ The limited collection and reporting of baseline socioeconomic characteristics was evident in our review: of the original trials of interventions included in this review, none presented information on individual socioeconomic position and only two reported on educational attainment and ethnicity of trial participants (Supplementary Data Table S3).^18–27^^,^^36^^,^^76–78^

The most comprehensive body of research to date has been on therapist-delivered interventions for low back pain, spanning subgroup analysis of the BeST trial,^53^ systematic review and narrative syntheses^79^^,^^80^ and IPD meta-analysis of up to 19 trials with a total of 9328 participants and using several novel methodological approaches.^81^^,^^82^ The available data from these trials permitted consideration of only some of the PROGRESS-Plus social stratifiers, mainly age and sex. They concluded from their analyses that ‘there is very little clinical effectiveness or cost-effectiveness justification for using the baseline characteristics we studied to define groups who might benefit from different back pain treatment’.^81^ A recent equity-focussed systematic review of chronic disease self-management support interventions also found little evidence on differential effects from trials, but noted that observational studies suggested lower participation rates among lower SES.^83^ From limited data presented, we found no strong evidence of selective non-participation or drop-out from the original trials of interventions in the current review (Supplementary Data Table S4).^18–27^^,^^36^^,^^76–78^^,^^84^

### What this study adds

Our review extends previous research to specifically consider equity issues and provide a critical synthesis of currently available evidence on differential effectiveness for the range of interventions recommended in the ROI tool. Despite the high burden of musculoskeletal conditions there has been surprisingly little focus on equity when investigating the effects of interventions. Given the well-recognized challenges in obtaining rigorous evidence on equity effects from quantitative analysis of single and multiple trials, our review underscores the importance of embedding equity considerations across the research cycle including intervention development and process evaluation. While none of the interventions in our review was designed deliberately to be ‘equity focussed’, they have demonstrated overall effectiveness in populations with varying degrees of social diversity and many are now already at fairly advanced stages of implementation. At the time of this review almost 1300 health professionals had been trained to deliver ESCAPE-pain. Prior to COVID-19, it was offered at 295 NHS facilities and leisure/community centres across UK, including some in more deprived neighbourhoods. In total, 19 300 people have taken the programme and an online version has been released (personal correspondence, M. Hurley, I. Rodrigues de Abreu).^85^ Over 11 000 people had registered for the 6-week free, online BeST training programme aimed at health professionals and accredited by the British Psychological Society.^86^ Trained therapists covered at least 177 NHS Trusts across the UK (personal correspondence, S. Lamb). An estimated 500 qualified, experienced yoga teachers across England had undergone a 300-hour training programme to deliver the Yoga for Healthy Lower Backs programme (personal correspondence, A. Trewhela).^87^ Over 500 physiotherapists had received training to deliver the STarT Back intervention. Twelve Academic Health Science Networks supported its provision across UK (personal correspondence, L. Campbell, N. Evans).^88^ A national mobilization plan is rolling out First Contact Practitioner services for musculoskeletal care with the objective of ensuring that all patients in UK will have direct access to this service, typically physiotherapists, by 2023/24. Over 54 300 appointments took place across 32 Sustainability & Transformation Partnerships over the 11-month pilot phase.^89^

### Limitations

We used a citation search strategy that will tend to miss relevant studies of other similar interventions. We restricted our attention to studies with an appropriate comparison of effect between social groups. There may be other studies, including those excluded at title/abstract stage, which include useful information or discussion on equity-related matters in relation to these or similar interventions, but we think it unlikely that we missed additional rigorous evidence of effect moderation. For example, one systematic review^90^ and four original research articles^91–94^ reported the characteristics of patients accessing physiotherapy direct access/self-referral schemes relative to other services, but since they reported no data on outcomes by social stratifier, they were excluded from the current review.

## Conclusions

We found no highly credible evidence against the assumption that seven interventions recommended for the prevention and management of musculoskeletal health are equally effective in different social groups. A policy of restricting or targeting these interventions to subpopulations is not supported. Most of these interventions are already being actively implemented, many achieving substantial reach nationally. Equity concerns may be best served by investing in equity-focussed action aimed at ensuring fair access to, and participation in, these interventions. Routine collection of key social variables during implementation should underpin this.

## Supplementary Material

Effects_on_health_inequalities_PHE_systematic_review_SUPPLEMENT_fdac014

## Notes

**G. Peat**, Professor of Clinical Epidemiology

**K.P. Jordan**, Professor of Biostatistics

**R. Wilkie**, Senior Lecturer in Public Health & Epidemiology

**N. Corp**, Post-doctoral Research Fellow: Information Science/Systematic Reviews

**D.A. van der Windt**, Professor of Epidemiology

**D. Yu**, Senior Research Fellow: Chronic Disease Surveillance

**G. Narle**, MSK Policy Implementation Manager

**N. Ali**, National MSK Lead
