# Supplementary material for: Do recommended interventions widen or narrow inequalities in musculoskeletal health? An equity-focussed systematic review of differential effectiveness
Source: J Public Health (Oxf). 2022 Mar 6;44(3):e376–87. doi: 10.1093/pubmed/fdac014 (PMC9424108; doi:10.1093/pubmed/fdac014)
Supplement: Effects_on_health_inequalities_PHE_systematic_review_SUPPLEMENT_fdac014 [file effects_on_health_inequalities_phe_systematic_review_supplement_fdac014.docx]

**Supplementary Data**

**Table S1.** Parent publications and intervention description for interventions recommended in Public Health England's Return on Investment tool for musculoskeletal health

| **Intervention** | **Intervention content (from original RCTs)** |
| --- | --- |
| Cognitive and Psychological Approaches (CBT) including Exercise | ***Johnson et al.*** Parent publications^18^  **Setting:** General practice/community. **Providers:** Physiotherapists with 4 training days. **How:** group discussion, the use of case vignettes, and practical (physical) activities delivered in groups of 4-10 (+Mailed educational pack containing a booklet and audio-cassette) and weekly homework to practice skills and take part in behavioral experiments at home. **Manual/protocol:** ?no. **No. of sessions:** 8. **Duration of intervention:** 6 weeks. **Duration of sessions:** 2 hours. **Intervention supporting materials:** - |
|  | ***Lamb et al.*** Parent publications^19,27,28^  **Setting:** General practice/community. **Providers:** Physiotherapists, occupational therapists, psychologists, nurses with 2-day training. **How:** Cognitive behavioural intervention targeted behaviours and beliefs about physical activity and avoidance of activity delivered to groups of 8 (mean) plus individualised home exercise programme. **Manual/protocol:** Yes. **No. of sessions:** 6 (+1). **Duration of intervention:** 6 weeks. **Duration of sessions:** 1.5 hours. **Intervention supporting materials:** Back Skills Handbook which included home tasks and patient education materials |
| STarT Back (Subgrouping for Targeted Treatment) | Parent publications^20,29^  **Setting:** General practice/NHS centres. **Providers:** Physiotherapists with 9 days additional training. **How:** Eligible patients completed STarT Back tool and stratified into low, medium, and high risk and offering matched treatment (advice/written info/video; standard physiotherapy; psychologically informed physiotherapy). **Manual/protocol:** Yes. **No. of sessions:** Mean 3.9. **Duration of intervention:** n/a. **Duration of sessions:** n/a. **Intervention supporting materials:** a pamphlet about local exercise venues and self-help groups, 15-min educational video, the Back Book. |
| Yoga for Healthy Lower Backs | Parent publications^21,30-32^  **Setting:** Non-medical centres. **Providers:** 20 experienced yoga teachers from the British Wheel of Yoga and Iyengar Yoga who attended program training sessions over 2 weekends. **How:** Class of <15 **Manual/protocol:** Yes - manualised class plan and student manual. **No. of sessions:** 12. (Participants were also encouraged to undertake yoga for 30 minutes daily or to practice at least 2 times per week, and to use the compact disc). **Duration of intervention:** 12 weeks. **Duration of sessions:** 75 min. **Intervention supporting materials:** Student manual, mat, relaxation compact disc, home practice sheets |
| ESCAPE-pain | Parent publications^22,33,34^  **Setting:** Physiotherapy outpatient department. **Providers:** Single experienced physiotherapist. **How:** Integrated patient education, with simple self-management and pain coping strategies followed by individualized progressive exercise program, delivered individually or to groups of 8. **Manual/protocol:** Yes - protocol. **No. of sessions:** 12. **Duration of intervention:** 6 weeks. **Duration of sessions:** 50-65 min. **Intervention supporting materials:** Not stated |
| PhysioDirect (Early telephone assessment and advice) | Parent publications^23,35,36^  **Setting:** Community physiotherapy services. **Providers:** Senior physiotherapists. **How:** GP referrals or self-referrals invited to telephone senior physiotherapist for initial assessment and advice, then sent written info, call back in 2-4wk, F2F if necessary or desired. **Manual/protocol:** Yes - for telephone assessment. **No. of sessions:** 47% managed by phone only; Mean F2F = 1.91. **Duration of intervention:** n/a. **Duration of sessions:** n/a. **Intervention supporting materials:** Training, assessment software and computerised templates, advice and exercise leaflets. |
| Self-Referral to Physiotherapy | ***Holdsworth et al.*** Parent publications^24^  **Setting:** General practices/physio services. **Providers:** GPs and NHS physiotherapy services. **How:** Self-referral to physiotherapy introduced as a new service in parallel to GP referral. New service publicised to the practice population over a 3-month period using patient newsletters, posters in the practice and by the primary care team verbally informing patients. **Manual/protocol:** No. **No. of sessions:** Median of 4 contacts. **Duration of intervention:** n/a. **Duration of sessions:** n/a. **Intervention supporting materials:** - |
|  | ***Mallett et al.*** Parent publications^25^  **Setting:** GP practices/physio MSK outpatients. **Providers:** GPs and NHS physiotherapy services. **How:** Self-referral to physiotherapy introduced as a new service in parallel to GP referral. SR pathway education for GPs, practice managers and primary care staff was undertaken. Patient information leaflets and SR advertisements were placed in all participating GP surgeries. SR patients underwent telephone triage before F2F appointment. **Manual/protocol:** No. **No. of sessions:** Mean 2.85 sessions. **Duration of intervention:** n/a. **Duration of sessions:** n/a. **Intervention supporting materials:** - |
| Vocational Advice in Primary Care | Parent publications^26,37^  **Setting:** General practices. **Providers:** 4 vocational advisors (physiotherapists) newly recruited to the role and given a 4.5 day training programme. **How:** Stepped care as required - telephone advice (step 1), 1 or more face-to-face meetings (step 2), contact with employers (step 3). **Manual/protocol:** No? **No. of sessions:** Median no of contacts = 2 (mostly by telephone). **Duration of intervention:** Unclear (mostly 1-2 contacts only). **Duration of sessions:** Median of 13 min for telephone advice; 60 mins for F2F. **Intervention supporting materials:** - |
| F2F Face-to-face; GP General practice; LBP Low back pain; MSD Musculoskeletal Disorder; OA Osteoarthritis | |

**Table S2.** Web of Science citation reference search

| **Parent paper** | **Number of Citations*** | **Total unique** |
| --- | --- | --- |
| Johnson, R. E., et al. *Spine (Phila Pa 1976) 2007;***32**(15): 1578-1585. | 1 + 129 (121 unique) = 121 unique | 121 |
| Lamb, S. E., et al. *Lancet 2010;* **375**(9718): 916-923. | 242 (233 unique) + 1 + 1 = 235 unique | 311 |
| Lamb, S. E., et al. *Health Technol Assess 2010,* **14**(41): 1-253, iii-iv. | 80 (79 unique) +1 + 2 + 2 + 3 = 84 unique |  |
| Lamb, S. E., et al. *Pain 2012;* **153**(2): 494-501. | 31 (30 unique) = 30 unique |  |
| Hurley, M. V., et al. *Arthritis Care Res (Hoboken) 2012,* **64**(2): 238-247. | 99 (98 unique) = 98 unique | 235 |
| Hurley, M. V., et al. *Arthritis Rheum 2007;* **57**(7): 1211-1219. | 140 (135 unique) = 135 unique |  |
| Hurley, M. V., et al. *Arthritis Rheum 2007,* **57**(7): 1220-1229. | 56 (53 unique) = 53 unique |  |
| Salisbury, C., et al. *BMC Health Serv Res 2009;* **9**: 136. | 11 unique | 89 |
| Salisbury, C., et al. *Health Technol Assess 2013;* **17**(2): 1-157, v-vi. | 36 (34 unique) + 31 + 1 + 1 = 66 unique |  |
| Salisbury, C., et al. *BMJ 2013;* **346**: f43. | 1+ 3 + 1 + 22 (1 unique) = 26 unique |  |
| Holdsworth, L. K., et al. *Physiotherapy 2007;* **93**(1): 3-11. | 54 = 51 unique | 56 |
| Mallett, R., et al. *Musculoskeletal Care 2014,* **12**(4): 251-260. | 11 unique |  |
| Hay, E. M., et al. *BMC Musculoskelet Disord 2008;* **9**: 58. | 90 (86 unique) + 1 = 86 unique | 706 |
| Hill, J. C., et al. *Lancet 2011;* **378**(9802): 1560-1571. | 672 (648 unique) + 1 + 1 + 1 + 2 (1 unique) + 1 = 652 unique |  |
| Bishop, A., et al. *BMC Musculoskelet Disord 2014;* **15**: 232. | 12 unique | 19 |
| Wynne-Jones, G., et al. *Pain 2018;* **159**(1): 128-138. | 10 unique |  |
| Cox, H., et al. *Complement Ther Clin Pract 2010;* **16**(2): 76-80. | 12 (10 unique) = 10 unique | 106 |
| Cox, H., et al. *Complement Ther Clin Pract 2010;* **16**(4): 187-193. | 32 (28 unique) = 28 unique |  |
| Tilbrook, H. E, et al. (2011). Yoga for chronic low back pain: a randomized trial. *Ann Intern Med 2011;* **155**(9): 569-578. | 108 (98 unique) = 98 unique |  |

*each paper may be associated with more than one relevant record in the database

**Total references identified =** 1817 (sum of ‘unique’ references for each individual paper)

**‘Total unique’ references identified =** 1643 (sum of unique references for each group of papers)

**Duplicates =** 163 (‘total unique’ references across all groups)

**Total unique references =**1480 (across all groups/papers)

**Table S3.** PROGRESS Plus characteristics of participants in the original trials

| **Intervention** | **n** | **Place of residence** | **Race, ethnicity, culture** | **Occupation** | **Gender/sex** | **Education** | **Socioeconomic position** | **Age*** |
| --- | --- | --- | --- | --- | --- | --- | --- | --- |
| CBT, including exercise^18^ | 234 | E. Cheshire | Nr | In employment = 67%; Routine/manual occupation = 28% | Female: 60% | nr | nr | Age: 48 (11) |
| BeST^19,27^ | 701 | Norwich, Coventry & Solihull, S. Warwickshire, Birmingham, Langbaurgh, N. Warwickshire | Mixed/Asian/Black/Chinese = 1%/4%/2%/<1% | In employment = 50% | Female = 60% | Left FT education aged≤16 = 55% | Received benefit payments = 40% | Age: 54 (15) |
| STarT Back^20^ | 851 | N. Staffordshire, Stoke-on-Trent | Nr | In employment = 62%; Routine/manual occupation = 51% | Female = 59% | nr | nr | Age: 50 (15) |
| PhysioDirect^23,36^ | 2249 | Bristol, Somerset, Stoke-on-Trent, Cheshire; Living in most deprived neighbourhoods† = 19% | Mixed/Asian/Black/Chinese/Other = 1%/1%/1%/<1%/<1%; Native language not English = 3% | In employment = 60%; Elementary occupation = 10% | Female = 59% | nr | nr | Age: median 48 (IQR 36-62) |
| Self-referral: Holdsworth^24,76-78^ | 3010 | 29 locations across Scotland; Urban/semi-rural/rural = 41%/45%/15%; Living in more deprived neighbourhoods‡ = 32% | Nr | In employment = 58% | Female = 60% | nr | nr | Age: 53 (16) |
| Self-referral: Mallett^25^ | 194 | Barnsley | Nr | nr | nr | nr | nr | nr |
| Yoga for Healthy Lower Backs^21^ | 313 | Cornwall, N. & W. London, Manchester, York | Nr | In employment = 78%; Other employee = 58% | Female = 70% | Left FT education aged ≤16 = 34% | nr | Age: 46 (11) |
| **Intervention** | **n** | **Place of residence** | **Race, ethnicity, culture** | **Occupation** | **Gender/sex** | **Education** | **Socioeconomic position** | **Age*** |
| ESCAPE-pain^22^ | 418 | SE London | Nr | nr | Female = 70% | nr | nr | Age: 66 (range 50-91) |
| Vocational advice in primary care^26^ | 338 | W. Midlands | Nr | In employment = 69%; Routine occupation = 20% | Female = 58% | nr | nr | Age: 49 (10) |
| BeST Back Skills Training; ESCAPE-pain Enabling Self-management and Coping with Arthritic Knee Pain through Exercise; nr not reported  No studies reported on religious or social capital characteristics so these are not displayed.  † most deprived quintile of Index of Multiple Deprivation; ‡ Carstairs index 5-7; expressed as combined group mean and standard deviation unless otherwise stated; * Mean (SD) unless otherwise stated | | | | | | | | |

**Table S4.** Selective participation and treatment compliance/drop-out by PROGRESS Plus characteristics in the original trials for PHE RoI interventions

| **Intervention** | **Participation** | **Treatment adherence/drop-out** |  |
| --- | --- | --- | --- |
| CBT, including exercise^18^ | “a few small differences between patients who did and did not participate in the study, but…no major threats to external validity” Consenters at the initial GP recruitment stage older and included more females than non-consenters; females less likely to attend a pretrial assessment. | - |  |
| BeST^19,27^ | “There were no important differences between those participants who were determined eligible and randomised, and those who were determined eligible and were not randomised.” | See Knox et al^55^ for analysis of treatment compliance  “Follow-up was 85% at 12 months, with no significant difference between groups. Participants who provided data at 12 months did not differ from those who were lost to follow-up in terms of baseline characteristics” |  |
| STarT Back^20^ | - | ”Baseline sex, disability, and duration of pain were similar for participants with data at 12 months and those lost to follow-up. However, those lost to follow-up were younger: mean age of the individuals who responded at 12 months was 52·0 years [SD 14·2] compared with 42·3 years [14·0] for those who did not respond”. |  |
| PhysioDirect^23,36^ | Of eligible patients, those randomised were younger. No differences for sex or deprivation | Among patients randomised to PhysioDirect intervention, no differences in age and sex observed between those receiving F2F appts vs telephone alone |  |
| Self-referral: Holdsworth^24,76-78^ | - | - |  |
| Self-referral: Mallett^25^ | - | - |  |
| Yoga for Healthy Lower Backs^21,84^ | - | - |  |
| ESCAPE-pain^22^ | “Although it was slightly more difficult to recruit participants into Grp-rehab, there was no evidence of differential recruitment to trial arms | “By 6 months, 76 (18%) participants had withdrawn (Figure 1). There was no evidence of differential attrition.” |  |
| Vocational advice in primary care^26^ | - | - |  |
| BeST Back Skills Training; ESCAPE-pain Enabling Self-management and Coping with Arthritic Knee Pain through Exercise | | | |
